# 756. Factors that Increase Engagement on Social Media for an Infectious Diseases Fellowship Program

**DOI:** 10.1093/ofid/ofad500.817

**Published:** 2023-11-27

**Authors:** John Cherian, Erica Herc, Nicholas F Yared, Indira Brar, Smitha Gudipati, Anita Shallal

**Affiliations:** Henry Ford Health, Detroit, Michigan; Henry Ford Hospital, Detroit, Michigan; Henry Ford Health System, Detroit, Michigan; Henry Ford Hospital, Detroit, Michigan; Henry Ford Health System, Detroit, Michigan; Henry Ford Health, Detroit, Michigan

## Abstract

**Background:**

Social media is increasingly being used among Infectious Disease (ID) programs. It can be a valuable recruitment tool in addition to providing education, self-promotion, and increasing collaborations across the ID community. We sought to analyze factors associated with increased engagement for our ID fellowship program’s Twitter account.

**Methods:**

We analyzed various metrics from consecutive Twitter posts and utilized the Twitter Analytics Dashboard to extract data from time of account inception. Impressions were defined as the number of times a tweet was seen by users, and engagements as a composite of interactions with a tweet. Tweet content was organized into educational, social, promotional, and other categories. Educational content was subcategorized into tweetorial, microbiology, question & answer (Q&A), and journal articles. A subset of tweets were analyzed for engagement from April 2022 to March 2023 for trends according to content and date the tweet was posted. Standard univariate descriptive statistical analyses, moods median test, and one-way analysis of variance was performed. Data analysis was performed using R version 3.4.0.

**Results:**

460 tweets were posted from October 2020 to March 2023 (Figure 1). Educational subcategories showed a large amount of engagement related to microbiology, with specifically more retweets, likes, and profile clicks (Table 1). There was no difference in engagement based on time of day of tweet (Table 2), although profile clicks were significantly more likely if tweets occurred on a weekend (p=0.04). A subset of 284 tweets from April 2022 to March 2023 revealed that months where multiple content categories were tweeted resulted in the most engagement (Figure 2). Generally, more twitter posts correlated with more engagement with a Pearson correlation coefficient of 0.7.

**Figure 1.**
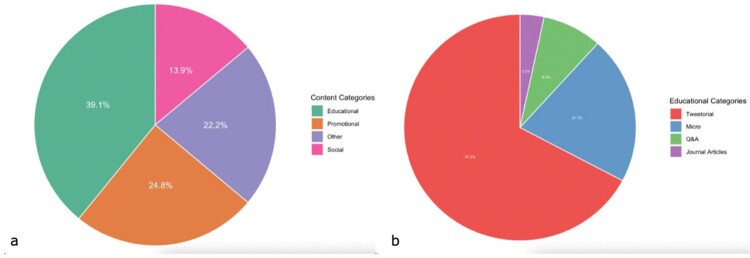
Tweet content by (a) overall categories and (b) educational categories.

**Figure:**
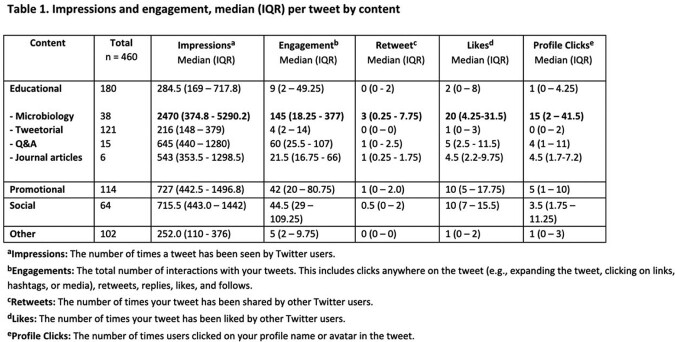

**Figure:**
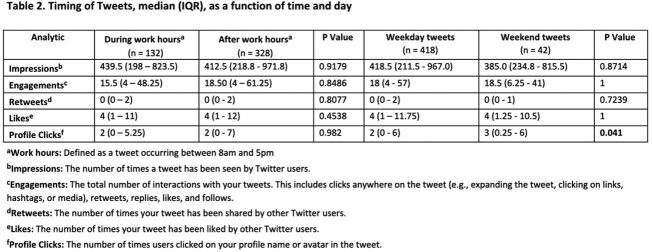

**Conclusion:**

To increase engagement with social media, there should be frequent tweets with varied content categories. Subcategories of content such as microbiology tweets draw further interest and showcase a program's educational and clinical innovation.

**Figure 2.**
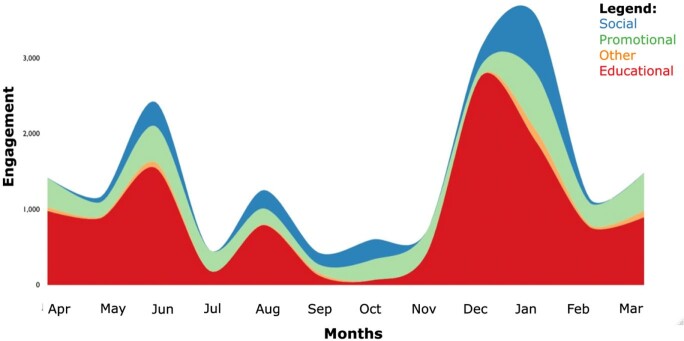
Composite categorical engagement from April 2022 – March 2023.

**Disclosures:**

**Indira Brar, MD**, Gilead: Advisor/Consultant|Gilead: Grant/Research Support|Gilead: Honoraria|Janssen: Grant/Research Support|Janssen: Honoraria|ViiV: Advisor/Consultant|ViiV: Grant/Research Support|ViiV: Honoraria

